# Subclinical Hypothyroidism in Children and Adolescents: Is It Clinically Relevant?

**DOI:** 10.1155/2015/691071

**Published:** 2015-03-29

**Authors:** Aneta Gawlik, Kamila Such, Aleksandra Dejner, Agnieszka Zachurzok, Aleksandra Antosz, Ewa Malecka-Tendera

**Affiliations:** ^1^Department of Pediatrics, Pediatric Endocrinology and Diabetes, School of Medicine in Katowice, Medical University of Silesia, 40752 Katowice, Poland; ^2^Medical Students' Scientific Association, 40752 Katowice, Poland; ^3^Department of Pediatrics, Pediatric Endocrinology and Diabetes, Upper-Silesian Pediatric Health Center, 40752 Katowice, Poland

## Abstract

Although subclinical hypothyroidism (SH) is a common clinical problem, its diagnosis tends to be incidental. According to the definition, it should be asymptomatic, only detectable by screening. The presence or coincidence of any symptoms leads to L-thyroxine treatment. The clinical presentation, especially in younger patients with subclinical hypothyroidism, is still under dispute. Accordingly, the aim of this paper was to review the literature from the past seven years. The literature search identified 1,594 potentially relevant articles, of which 24 met the inclusion criteria. Few studies focus on the symptomatology of subclinical hypothyroidism, and most of them analyzed a small number of subjects. A significant correlation was found by some authors between subclinical hypothyroidism and a higher risk of hypertension, dyslipidemia, and migraine. No evidence of the impact of subclinical hypothyroidism on weight, growth velocity, and puberty was revealed. As the quality of most studies is poor and no definite conclusions can be drawn, randomized, large-scale studies in children and adolescents are warranted to determine the best care for patients with SH.

## 1. Introduction

From the biochemical point of view, subclinical hypothyroidism (SH) is characterized by mildly elevated serum TSH concentrations, with normal concentrations of serum free and total triiodothyronine (T3) and thyroxine (T4), without the typical symptoms of thyroid disease. SH prevalence in adults ranges from 4 to 10% [1]. In the pediatric population, the prevalence of this thyroid disorder is estimated to be less than 10% [2]. According to Biondi and Cooper, children with SH can present some minimal or nonspecific signs and symptoms [1]. There is a paucity of long-term prospective research studying the natural history of subclinical hypothyroidism and its consequences in childhood [3, 4]. A preliminary SH diagnosis is confirmed by laboratory test when TSH concentration is above the statistically defined upper limit of the reference range [5].

In the adult population with subclinical thyroid disease, SH is associated with a risk of progression to overt thyroid disease, lipid disorders, increased risk of atherosclerosis, and mortality due to cardiovascular diseases [6]. The public data regarding the clinical manifestation of SH in children and adolescents are inconsistent as most papers indicate SH to be asymptomatic [7].

Accordingly, the aim of this paper was to analyze studies reporting signs and symptoms presented by children and adolescents diagnosed with subclinical hypothyroidism.

## 2. Methodology

### 2.1. Literature Search

In order to identify studies evaluating the clinical manifestations and symptoms of subclinical hypothyroidism in children, a systematic PubMed literature search was conducted. The search terms used in the medical subject headings (MeSH) included (subclinical [All Fields] AND (“hypothyroidism” [MeSH Terms] OR “hypothyroidism” [All Fields])) as well as (subclinical [All Fields] AND (“hypothyroidism” [MeSH Terms] OR “hypothyroidism” [All Fields]) AND (“infant” [MeSH Terms] OR “child” [MeSH Terms] OR “adolescent” [MeSH Terms])) and SH hypothyroidism [All Fields] AND “humans” [MeSH Terms] AND (“infant” [MeSH Terms] OR “child” [MeSH Terms] OR “adolescent” [MeSH Terms]) and hyperthyrotropinemia [All Fields] AND “humans” [MeSH Terms] AND (“infant” [MeSH Terms] OR “child” [MeSH Terms] OR “adolescent” [MeSH Terms]). These terms were combined in various ways to generate a wider search.

**Figure 1:**
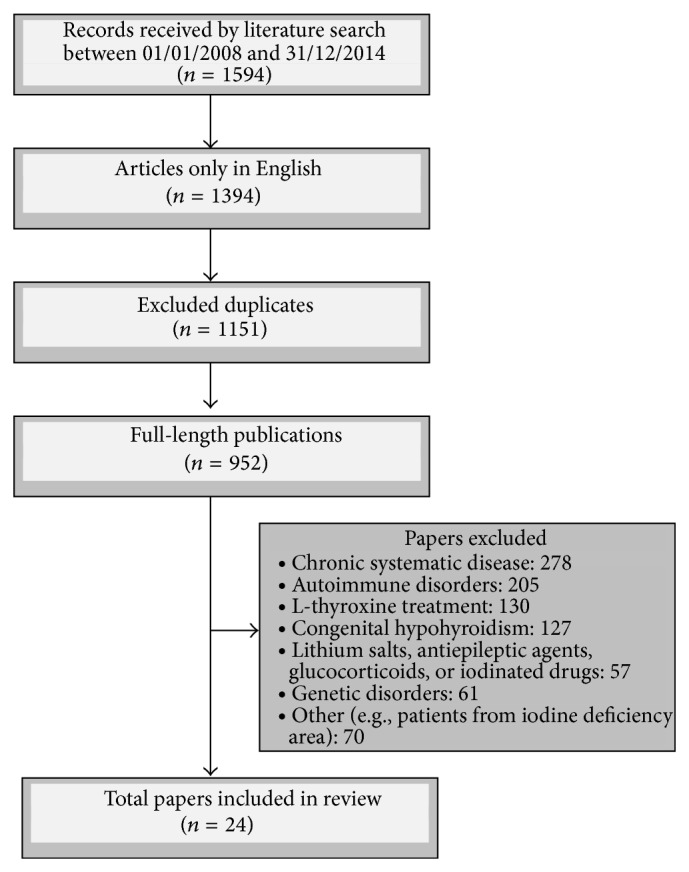
Study design and inclusion/exclusion criteria.

In addition, the references of selected articles were checked with a view to identify papers not detected by our search strategy. Only full-length publications that met the following criteria were included: (1) long-term prospective or retrospective studies regarding the clinical signs of SH in the pediatric cohort; (2) only studies in English; (3) studies published between January 2008 and December 2014. Exclusion criteria were the following: (1) studies including patients with chronic systematic diseases, genetic syndromes, or autoimmune disorders or under concomitant therapy with lithium salts, antiepileptic agents, glucocorticoids, or iodinated drugs; (2) studies regarding the treatment and effects of L-thyroxine replacement therapy. At least two authors independently selected articles for inclusion and exclusion criteria (Figure 1).

## 3. Results

After reviewing 1,594 titles, abstracts, and full-length texts, 24 articles meeting the inclusion criteria were selected for the final analysis [8–31]. There were sixteen cross-sectional studies [9, 10, 12–15, 17–19, 21–27, 29, 30], twelve studies that met the criteria of case-control trials [9, 11, 14, 18–20, 23–25, 27, 29, 30], ten longitudinal trials [9–15, 17, 21, 23, 25], and four retrospective studies [8, 16, 28, 31]. Overall, data from a total of 139 756 children were reported. Most studies included small numbers of children; only one retrospective study described a large population [8]. The subjects' ages ranged from 2 weeks to 18 years, whilst the follow-up after SH diagnosis was from 2 weeks to 108 months. The cut-off limits for TSH concentrations were between 3.0 and 6.7 mIU/L and ≤10 mIU/L. Studies analyzing the clinical manifestations of SH are demonstrated in Table 1.

### 3.1. SH and Weight/BMI

In their cross-sectional controlled long-term study of 36 children with SH and controls, Cerbone et al. [9] assessed the relationship between weight/BMI and persistent untreated SH during follow-up. The authors concluded that only three (8%) SH children were obese. No significant alterations in weight or BMI were noticed during the period from diagnosis to study enrollment (3.3 ± 0.3 yrs). Similar observations concerning stable BMI values were presented by Wasniewska et al. [10].

In a case-control study, Kuiper and van der Gaag [11] demonstrated the impact of a diet rich in iron, iodine, and vitamin A. The authors hypothesized that the main problem regarding SH in children was an immunological dysfunction or deficiency of micronutrients, which cause disturbed thyroid hormone production. The study encompassed 54 children with SH, aged 1–14 yrs, divided into 2 groups: diet and control. The 3-month diet consisted of green vegetables, beef, full fat milk, and butter. TSH, FT4, and BMI were evaluated during the follow-up. The results showed a significant drop in TSH in the diet group. The limitations of the study were a small number of patients and a nonrandomized study design.

Some studies have confirmed the relationship between the levels of TSH and FT3 and change of body weight. The level of FT4 remained stable during follow-up. Leptin was hypothesized to link the weight status with TSH. This would suggest that TSH and FT3 concentrations in obese subjects are a consequence rather than the cause of obesity [12–15].

Reinehr et al. [12] analyzed thyroid hormones in female adolescents with obesity and anorexia nervosa before and after weight normalization. The authors determined the serum levels of TSH, FT3, and FT4 in 100 obese girls, 32 normal-weight girls, and 20 girls with anorexia nervosa aged 14–18 yrs. The measurements were carried out at baseline and after 1 year. Additionally, leptin, insulin, and homeostasis model assessment (HOMA) index were analyzed in obese and normal-weight girls in order to determine the degree of insulin resistance. The results revealed that, compared to normal-weight girls, the levels of TSH and FT3 were significantly lower in girls with anorexia nervosa and significantly higher in the obese. The obese patients who lost more than 5% of their weight demonstrated a valid decrease in FT3 and TSH, whilst patients with anorexia nervosa who gained weight showed a notable increase in FT3 and TSH. Unlike leptin, HOMA and insulin were not correlated to TSH, FT3, and FT4.

Similar conclusions were drawn by Baş et al. [13], who observed a positive correlation between weight loss and normalization of serum TSH levels.

The study by Grandone et al. [14] encompassed 938 obese children and adolescents (450 females). The aims of the study were to (1) ascertain the spread of abnormally elevated TSH levels in young obese Italian patients, (2) determine if highly elevated levels of TSH in obese children could constitute cardiovascular and metabolic risk factors, and (3) to check if elevated TSH is reversible after weight loss. Anthropometric, metabolic, and hormonal variables were determined at baseline and, in a subgroup of children with hyperthyrotropinemia, after a six-month weight-loss program. The results suggest that (1) a moderate elevation of TSH concentrations is common in obese children, (2) hyperthyrotropinemia is not connected with metabolic risk factors in obese patients, and (3) moderate elevation of TSH is reversible after weight loss, and, according to the authors, no treatment is needed.

The association between TSH, FT3, FT4, and weight status, as well as their changes during and after a lifestyle intervention in obese children, was also studied by Wolters et al. [15]. They evaluated the weight status/BMI-SDS in 477 obese children who took part in a 1-year lifestyle intervention in a 2-year longitudinal study. At baseline, 39% of the children had TSH level >3.0 mIU/L. A reduction in BMI-SDS (>0.5) during the intervention was associated with a decrease and normalization of TSH and FT3 concentrations. Interestingly, a decrease in TSH and FT3 concentrations by lifestyle intervention was at the same time associated with an increase in BMI-SDS after the intervention time.

Two other studies investigating the correlation between BMI in obese children and hyperthyrotropinemia have produced interesting findings. Hari Kumar et al. [16] compared the TSH level in a cohort of 50 children divided into two groups: overweight and obese. The TSH levels were comparable in both groups and the authors did not find a significant impact of the degree of obesity on the level of serum TSH. Shalitin et al. [17] investigated the potential relationship between the levels of serum TSH and other metabolic and hormonal variables before and after weight loss. They enrolled 207 obese children and adolescents aged 5–18 yrs who underwent biochemical, hormonal, anthropometric, and metabolic examinations before and after weight reduction. Hyperthyrotropinemia was confirmed in 46 (22.2%) participants and correlated positively with higher triglycerides. The decrease in TSH concentration correlated positively with a reduction in waist circumference. No correlation between TSH and leptin levels was confirmed. All the patients had normal FT4 concentrations. The investigators raised the question of the necessity to treat hyperthyrotropinemia in obese children.

Radetti et al. [18] investigated the pathological structure/morphology of the thyroid gland and its function in obese children. The cross-sectional study included 186 overweight and obese young patients. Ultrasonography, FT3, FT4, TSH, and antithyroid antibodies were performed in all subjects. The control group was composed of 40 healthy children. The cohort was divided into four groups: A: with Hashimoto disease, B: with positive antibodies and normal ultrasound, C: with negative antibodies and ultrasound pattern suggestive of Hashimoto's thyroiditis, and D: with negative antibodies and normal ultrasound. Groups A and C had higher TSH levels than groups B and D and controls. The study showed that alterations in thyroid structure and function in obese patients are quite common. Obesity is often associated with a low-grade inflammatory state, which would explain the abnormal ultrasound of the thyroid gland in patients with large deposits of adipose tissue.

The multicenter study by Rapa et al. [19] was based on 88 children and adolescents with SH diagnosed by at least two determinations of TSH above the upper limit of the reference range. The prevalence of overweight/obesity in the study group was 28.4%. Overweight and obese patients had significantly higher serum TSH levels than patients with normal weight (7.4 versus 5.7 IU/mL); the highest values were found in patients with hypoechogenicity in thyroid ultrasonography (8.5 IU/mL). The multiple regression analysis showed that increased serum TSH concentrations (multiple *R* = 0.31; *P* = 0.02) were independently caused by both younger age and overweight/obesity status.

### 3.2. SH and Intellectual Outcomes

In a study that included 30 SH children and 36 controls, Cerbone et al. [9] evaluated verbal, performance, and full-scale intelligence quotient (IQ) scores as well as the children's psychological development. The intellectual outcomes were normal for the children's age and comparable to controls. There was no relationship between mean serum TSH concentrations during the observation or the persistence of SH and IQ scores, IQ subtests, age-appropriate child behavior checklist, and Children's Depression Inventory scores.

Ergür et al. [20] conducted a case-control study including 17 children with SH and 17 healthy children with TSH serum levels 7.2 ± 0.89 mUI/L versus 3.6 ± 0.89 mUI/L, respectively. Analyzing the cognitive functions, such as active/passive attention, maintaining attention, and response inhibition, the authors reported significantly lower scores in the SH group than in controls, both on the Digit Span subtest of the Wechsler Intelligence Scale for Children-Revised (WISC-R) and the Stroop subtests. No significant differences were found between the SH group and the healthy controls in verbal fluency and encoding tests.

### 3.3. SH Blood Pressure and Cardiovascular Risk Factors

In their cross-sectional trial, Chen et al. [21] investigated the correlation between serum TSH levels and blood pressure in Chinese school-aged children without overt thyroid disease. Of 880 enrolled children, 127 (14.4%) were diagnosed with subclinical hypothyroidism. During the 7-month follow-up, both systolic and diastolic blood pressure *Z*-scores were found to be higher in subjects with subclinical hypothyroidism than in euthyroid subjects; in males, the scores increased linearly with increasing TSH.

Ittermann et al. [22] evaluated the relationship between serum TSH concentrations, pulse, and systolic and diastolic blood pressure in 12,353 subjects divided into two groups: children (3–10 yrs) and adolescents (11–17 yrs). Subclinical hypothyroidism was observed in 5% of the study population and was associated with continuous values of systolic and diastolic blood pressure in children and adolescents. The results suggest that SH is associated with an increased risk of hypertension. Although the pathophysiological mechanism underlying this finding is not well understood, authors speculate that it may be due to endothelial dysfunction, left ventricular hypertrophy, or thickened arterial walls. No relationship was observed between Tanner stage IV/V in adolescents and TSH concentration. Furthermore, adolescents with SH had lower pulse rate than euthyroid subjects.

Cerbone et al. [23] in a cross-sectional and controlled study evaluated the clinical and biochemical cardiovascular risk factors in untreated children with mild SH (TSH serum concentration 4.5–10 mU/L). After two years of the follow-up, children with SH had significantly higher waist-to-hip ratio, triglycerides to high-density-lipoprotein ratio, and homocysteine level and lower high-density lipoprotein-cholesterol. The authors concluded that mild long-lasting SH may be associated with subtle proatherogenic abnormalities although these changes may not represent the early stage of atherogenesis.

Positive correlation between the components of the metabolic syndrome and serum TSH was also found in Chinese adolescents by Zhang et al. [24].

### 3.4. SH and Linear Growth, Bone Maturation

In their cross-sectional controlled long-term trial, Cerbone et al. [9] also analyzed growth in children with persistent untreated SH. The parameters of height and bone age/chronological age were within normal ranges at the beginning of the trial and did not deteriorate over several years of follow-up. Despite the normal mean height, a subgroup of 8/36 (22%) SH children had short stature. Six of them were within the target height range, thus suggesting familial short stature; the other two (5.5%) had stature below the target height with normal growth velocity, normal IGF1 concentration, GH peak after stimulation test within the reference range, and a delay of 2 years in bone maturation, indicating a possible constitutional delay of growth and puberty. None of the parameters deteriorated over several years of follow-up in SH children and controls.

di Mase et al. [25] in their cross-sectional prospective trial evaluated the possible effect of untreated SH on bone health in childhood. Two methods were used: dual-energy X-ray densitometry (DXA) and quantitative ultrasound (QUS). The authors recruited 25 subjects with SH and 25 randomly chosen age- and sex-matched healthy children as controls. All values of lumbar spine DXA and phalangeal QUS were within the normal range, both in patients and in controls, despite the long-term duration of untreated SH. The statistical analysis showed that neither TSH levels at the beginning of the study nor the duration of SH had a significant impact on bone mineral density (BMD), amplitude-dependent speed of sound (Ad-SoS), and bone transmission time *Z*-scores (BTT *Z*-scores).

### 3.5. SH and Migraine

One cross-sectional study conducted by Fallah et al. [26] assessed the monthly prevalence of headache in children with subclinical thyroid disease. The authors concluded that children aged between 5 and 15 yrs diagnosed with SH suffered more frequently from the migraines which were also lasting longer. The authors suggest checking the thyroid function in migraineur children and adolescents. These findings were not confirmed by Ekici and Cebeci [27] who detected subclinical hypothyroidism only in 5% of children with headache. They concluded that initial endocrinological evaluation or screening for SH in children suffering from migraine is unnecessary.

### 3.6. Natural Course of SH

Lazar et al. [8] presented the natural history of thyroid function tests over five years in a large pediatric cohort. The authors analyzed the survey demographic data, referral diagnoses, and laboratory results (TSH, FT4, and thyroid antibodies) of 121,052 children with SH aged 0.5–16 yrs. First, the authors determined the relative proportion of regular and irregular thyroid function results in the cohort. Next, they established the natural history of initial abnormal TSH levels in otherwise healthy children without any thyroid disease. Finally, they defined the number of patients at an increased risk for developing subsequent abnormal TSH levels. Normal initial TSH concentrations were observed in 96.5% of children; 0.2% had low TSH concentrations (0.35 mIU/L), 2.9% elevated (5.5 to 10 mIU/L), and 0.4% highly elevated (10 mIU/L). In girls, the determination of TSH serum level was performed more frequently than in boys. Moreover, the frequency of TSH testing increased with age. In the second TSH evaluation, TSH levels within the reference range were documented in 40%, 73.6%, and 78.9% of those whose basic serum TSH was highly elevated, elevated, and low, respectively, and in 97% of those with normal initial TSH. Predictive factors for a sustained highly elevated TSH were initial TSH > 7.5 mIU/L (*P* = 0.014) and female gender (*P* = 0.047). The conclusions were as follows: (1) initial normal or slightly elevated TSH levels are likely to remain normal or spontaneously normalize without treatment; (2) patients with initial levels higher than 7.5 mU/L, particularly girls, are at a greater risk for persistent abnormal TSH concentration.

Also Wasniewska et al. [10] in their prospective cross-sectional controlled trial estimated the natural course of idiopathic SH. The authors analyzed 92 young patients whose clinical status, thyroid function, and autoimmunity were checked after 6, 12, and 24 months. All of the patients were asymptomatic, with no changes in BMI and height in follow-up. The 2-year prospective study showed that 38 (41.3%) patients out of the entire cohort aged <15 years normalized their TSH values during follow-up, whilst, in 11 (12%), the thyroid function deteriorated as demonstrated by the increase in TSH values to 10–15 mU/L. Overall, the beneficial evolution was more manifested in pubertal patients than in prepubertal ones; it was not significantly affected by sex, FT4 levels, or familial antecedents of thyroid disease.

### 3.7. Thyroid Status in Puberty

In their cross-sectional controlled long-term study, Cerbone et al. [9] found that, in nine (six females; 25%) of the 36 SH children, puberty started and proceeded normally despite the persistence of untreated SH (2.2–7.5 yrs). Moreover, the final accomplished height of four of the children (two females) was more than adequate for the target height according to Tanner et al. [32]. In two (5.5%) SH children, a possible constitutional delay of growth and puberty was suspected due to stature below TH, with normal growth velocity, normal IGF1 concentration, GH peak after stimulation test within its reference range, and a delay of 2 years in bone maturation.

Rapa et al. [19] concluded that, in 19 (21.6%) out of 88 children with SH, serum TSH concentrations were not influenced by puberty status as determined by visual inspection according to the Tanner scale.

### 3.8. SH and Vitiligo

Cho et al. [28] investigated the relationship between vitiligo and thyroid gland dysfunctions. The authors retrospectively compared the thyroid function of 254 children with vitiligo and 122 healthy controls. The results showed that there was no significant disparity in the range of thyroid disease between healthy children and the cohort with vitiligo.

### 3.9. SH and TSH-R Gene Mutations

Rapa et al. [19] in their multicenter study made an attempt to establish not only the clinical characteristics and biochemical parameters but also TSH-R gene variations in children and adolescents with SH. A total of 19 known variations in TSH-R gene were found in the study population, of which eight were nonsynonymous mutations found in ten patients. Those patients had a 1.9-fold higher prevalence of positive family history of thyroid diseases than patients without any detected mutation.

### 3.10. SH in In Vitro Fertilization Children

Two case-control studies [29, 30] assessed the condition of the thyroid gland in in vitro fertilization (IVF) babies. Onal et al. [29] analyzed the TSH concentration in newborns at the age of 2–4 weeks. They examined 98 babies conceived after classic IVF, of whom 10 had elevated serum TSH level above 6.5 mUI/L and were diagnosed with SH. Thyrotropin-releasing hormone (TRH) tests were executed in all subjects, both in the study group and in controls. The control group consisted of 10 naturally conceived babies with euthyroid hyperthyrotropinemia. The peak of TSH response was measured at 20 min. TSH response to TRH was considered normal if the peak TSH level was between 5 and 25 mIU/L. A peak value above 25 mIU/L was considered exaggerated, whilst that below 5 mIU/L was considered suppressed. The baseline TSH levels were 8.08 ± 1.66 mUI/L in the study group and 7.55 ± 0.80 mUI/L in controls. At 20 min of the TRH test, the TSH serum concentration was significantly higher in the population of IVF newborns than in naturally conceived controls: 55.57 ± 14.31 mUI/L versus 19.37 ± 3.47 mUI/L.

Sakka et al. [30] studied 106 IVF children aged 4 to 14 yrs and 68 randomly selected non-IVF controls of the same age. The cut-off TSH serum concentration of 5.0 mU/L was a criterion for the second TSH determination and thyroid ultrasound. Children were considered to have persistent hyperthyrotropinemia if the TSH level was confirmed. Persistent elevations of circulating TSH were observed in seven IVF children and in none of the controls. The higher SH incidence in in vitro fertilization babies than in controls did not depend on birth weight, gestational age, small for gestational age and adequate for gestational age status, breastfeeding duration, gender, current age, BMI-SDS or pubertal status of IVF children, BMI, thyroiditis, maternal polycystic ovary syndrome (PCOS), gestational diabetes mellitus, parity, arterial hypertension during pregnancy, or smoking of their mothers.

### 3.11. Reference Range for TSH

Kratzsch et al. [31] observed 1,004 children divided into 3 age groups in order to determine the reference ranges for thyroid function tests in children and adolescents and to identify factors that may influence the limits of these intervals. Puberty was followed by a rise of TSH, FT3, and T3 levels. T4 and t-uptake were significantly higher in girls than in boys. The exclusion of children with increased TPO-Ab and TG-Ab had no significant influence on the lower and upper limit of the reference interval for TSH. The authors concluded that age, BMI-SDS, white blood cells count, and gender should be taken into account when diagnosing SH based on serum TSH concentration.

## 4. Discussion

The definition of SH is purely biochemical [1]. The establishment of the upper limit of normal TSH in different age groups poses a challenge [33]. Furthermore, there is no consensus about the TSH level at which treatment should be considered. In most of the analyzed publications, serum TSH over 4.2 mUI/L was used as SH cut-off point [8–11, 13, 14, 16, 19–21, 23–30]. L-thyroxine therapy was not administered in any of the cited studies. Screening for thyroid disorders has become more common in recent years, thus leading to SH being diagnosed more frequently, especially in younger subjects.

The analysis of SH-related symptoms was based on two different types of studies. In the first type, SH confirmation was sought as a result of symptoms/clinical presentation [12–18, 26–30]. In the second type, SH was diagnosed incidentally and only then followed by clinical assessment for typical manifestation [9–11, 19–25]. The natural history of thyroid function was described in two studies [8, 10]. The question arises whether SH actually causes symptoms or whether their presence is a coincidence. The answer may influence the decision regarding pharmacotherapy. Older SH patients can present cardiovascular risks, depressed systolic function, and left ventricular diastolic dysfunction and may report reduced exercise tolerance [34]. What about the clinical manifestation and consequences in younger populations?

We only analyzed articles in which subjects presented biochemically pure SH. The prevalence of SH in children and adolescents in the analyzed literature was between 2.9% and 14.1% [8, 21, 25] and could be modified by weight. In the obese and overweight population, the percentage of SH was significantly higher, reaching even more than 30% [15, 16], hyperthyrotropinemia being a consequence rather than the cause of a higher BMI. Studies analyzing height, growth velocity, and puberty did not confirm a negative influence of SH [9].

SH should be distinguished from physiological or transiently increased TSH in the serum, especially during the recovery phase from nonthyroidal illnesses or after subacute, painless thyroiditis. For this reason, serum TSH should be checked after 3–6 months [31]. This observation was confirmed by two studies examining the natural course of SH [8, 10]. One of them, a retrospective analysis of 121,052 children, indicated TSH above 7.5 mIU/L and female sex as predictive factors for fixed SH [8]. Spontaneous reversibility of elevated TSH was found in 41–73% of subjects [8, 10].

Rarely, higher serum TSH concentrations are seen in patients with TSH-receptor mutations causing mild TSH resistance. It can affect up to 0.6% of white population and should be suspected in positive family history of elevated TSH, not coexisting with thyroid autoimmunity [35].

## 5. Conclusions

Subclinical hypothyroidism is a common clinical problem. According to the definition, SH is asymptomatic and detectable only by screening. However, there are a lot of controversies concerning population screening for SH as the benefits of therapy are not proven. Single placebo-controlled studies in older populations confirmed that screening and treatment by L-thyroxine improve the quality of life in only 1% of individuals [36]. The symptoms of SH are nonspecific. In some cases, the rise in TSH concentration was found incidentally, and only then the symptoms were looked for; in other cases, nonspecific complaints lead to the assessment of TSH. In both, there is no certainty concerning the connection between higher TSH levels and the presence of symptoms.

Our study limitation is searching in PubMed base only and restricting the search to English language. Probably a systematic search across multiple databases would yield more results. However, as the quality of most studies is poor and no definite conclusions can be drawn, randomized, large-scale studies in children and adolescents are warranted to determine the best care for patients with SH.

## Figures and Tables

**Table 1:** Subclinical hypothyroidism in children and adolescents, clinical studies 2008–2014.

Authors and study design	Subjects	Methods	TSH concentration [mIU/L]	SH: TSH cut-off [mIU/L]	Duration of follow-up (months)	Main results
Lazar et al. [8]^R^	121,052 with SH aged 0.5–16 yrs	TSH, fT4, fT3, TPO-Abs, and TG-Abs	—	>5.5	60	(1) Initial TSH greater than 7.5 mIU/L and female gender constitute predictive factors for sustained SH; (2) the natural history of initial mild TSH elevations (5–7.5 mIU/L) in otherwise healthy children not treated with thyroid or antithyroid medication revealed spontaneous normalization of TSH values

Cerbone et al. [9]^CS,CCL^	36 with SH^SG^, 36 without SH^CG^	BMI, fasting TSH, FT4, TG, TPO-Abs, TG-Abs, IGF-1, UIE, BA/CA ratio, and intellectual evaluation	6.4 ± 0.3^SG^ 2.6 ± 0.2^CG^	>4.2	24–108	(1) Persistent SH in children not associated with alterations in growth, bone maturation, BMI, or cognitive function or other complaints that could be ascribed to SH even after several years without therapeutic intervention

Wasniewska et al. [10]^CSL^	92 (MA: 8.1 ± 3.0 yrs)	TSH, FT4, TPO-Abs, TG-Abs, thyroid USG, and BMI	6.1 ± 1.3 baseline	>5.0	24	(1) The natural course of TSH values in a pediatric population with idiopathic SH characterized by a progressive decrease over time; (2) the majority of patients (88%) normalized or maintained unchanged TSH; (3) TSH changes not associated with any changes in either FT4 values or clinical status or auxological parameters

Kuiper and van der Gaag [11]^CCL^	27^SG^ 27^CG^	FT4, TSH, gender, diet, age at presentation, use of medication, and BMI	5.7^SG^ 5.5^CG^	>4.2	36	(1) In the diet group, 74% of the patients normalized TSH; (2) TSH at the end: 3.5 mIU/L^SG^ and 4.9 mIU/L^CG^

Reinehr et al. [12]^CSL^	100 obese girls, 32 normal-weight girls, and 20 girls with anorexia nervosa	TSH, FT3, FT4, BMI, leptin, insulin, and HOMA; girls with AN enrolled in a psychotherapy and nutritional rehabilitation program, obese girls in the 1-year obesity intervention program Obeldicks	—	—	12	(1) TSH and FT3 levels of girls with AN significantly lower compared to TSH concentrations of normal weight girls; (2) TSH and FT3 levels of the obese girls significantly higher; (3) the 21 obese females with weight loss >5% demonstrated a significant decrease in FT3 and TSH; (4) the 9 adolescents with AN and weight gain >5% showed a significant increase in FT3 and TSH; (5) insulin and HOMA not significantly correlated to TSH, FT3, and FT4; (6) leptin was correlated to TSH and FT3 in both cross-sectional and longitudinal analysis

Baş et al. [13]^CSL^	150 obese children (BMI > 95th pc)	TSH, FT3, FT4, TPO-Abs, TG-Abs, BMI, glucose, insulin, HOMA-IR, and weight reduction intervention	2.8 ± 1.4	>4.2	6	(1) At baseline, 23 (15.3%) subjects had elevated TSH, and 21 of these patients completed the weight reduction intervention; (2) out of 23 patients, 14 had a substantial weight loss and a significant decrease in TSH and FT3 level

Grandone et al. [14]^CS,CCL^	938 obese children and adolescents (BMI > 95th pc)	BMI, waist circumference, SBP, DBP, TG, HDL, insulin, glucose, TSH, FT3, FT4, TG-Abs, TPO-Abs, and weight loss program	5.1 ± 1.0^SG^ 2.3 ± 0.8^CG^	>4.2	6	(1) Isolated hyperthyrotropinemia diagnosed in 120 patients (62 girls) of the remaining 938 patients enrolled (12.8%); (2) in obese children, the increase in TSH is not associated with metabolic risk factors; (3) hyperthyrotropinemia is reversible after weight loss and these data suggest that it should not be treated; (4) BMI *Z*-score and FT3 levels were significantly higher in patients with elevated TSH, while their age was significantly lower

Wolters et al. [15]^CSL^	477 obese children (BMI > 97th pc)	TSH, FT3, FT4, BMI, and BMI-SDS	3.0 ± 1.4	>3.0	24	(1) 39% of the children demonstrated TSH levels above 3.0 mIU/L for TSH. (2) BMI-SDS reduction during the lifestyle intervention associated with a reduction in TSH and FT3 concentrations. (3) Moderately increased TSH and FT3 concentrations in obese children normalized in substantial weight loss

Hari Kumar et al. [16]^R^	1: overweight (*n* = 20) (BMI: 85th–95th pc); 2: obesity (*n* = 30) (BMI > 95th pc)	TSH, FT3, FT4, and BMI	3.22 ± 3.12 (overweight) 3.63 ± 2.24 (obesity)	>4.5	—	(1) Elevated TSH level (between 4.5 and 10 mIU/L) seen in 4/20 overweight and 9/30 of obese children; (2) the mean TSH comparable in both the groups; (3) no correlation between TSH and BMI; (4) the preliminary data did not show any relation between severity of obesity and TSH level

Shalitin et al. [17]^CSL^	207 obese children (BMI > 95th pc)^SG^	TSH, FT3, FT4, age, BMI, waist circumference, TG, TC, LDL, HDL, glucose, insulin, CRP, IL-6, homocysteine, adiponectin, leptin, ghrelin, and 12-week weight loss program	5.08 ± 0.84 (boys) 5.20 ± 1.31 (girls)	>4.0	24	(1) At baseline, 46 participants (22.2%) had hyperthyrotropinemia; (2) baseline TSH significantly correlated with TG levels but not with age, anthropometric, or other laboratory variables; (3) Of the 142 participants who completed the intervention, 27 (19%) had hyperthyrotropinemia; (4) no significant relationship between changes in TSH level and changes in BMI-SDS; (5) a significant correlation was found between the final TSH level and TG level and between the decrease in TSH level and the decrease in waist circumference

Radetti et al. [18]^CS,CC^	186 screened for thyroid disease^SG^, 40 without SH^CG^	TSH, FT3, FT4, TPO-Abs, TG-Abs, thyroid USG, percent of body fat, height-SDS, and BMI-SDS	A: 7.3 ± 10.6 B: 2.2 ± 1.4 C: 3.9 ± 2.0 D: 2.7 ± 1.7 Control: 2.1 ± 0.7 All: 2.6 ± 4.3	>3.6	—	(1) 23 children (12.4%) Abs (+) and an USG pattern-> Hashimoto's thyroiditis (group A); (2) 20 (10.8%) Abs (+) and normal USG (group B); (3) 70 subjects (37.6%) Abs (−) and an USG pattern like in Hashimoto's thyroiditis (group C); (4) 73 children (39.2%) Abs (−) with normal USG (group D); (5) groups A and B excluded due to diagnosed thyroiditis; (6) TSH serum levels significantly different in children with or without thyroid alterations at USG; (7) obese children may show a different degree of thyroid impairment

Rapa et al. [19]^CS,CC^	88	Height, weight, family history of thyroid diseases, thyroid USG TSH, FT3, FT4, UIE, and genetic variations in the TSH-R gene	Overweight/obese versus normal-weight patients (7.4 versus 5.7) Overweight/obese with hypoechogenicity versus patients with normal USG pattern (8.5 versus 6.8)	>4.5	1 visit	(1) TSH levels tended to be higher in the 20 patients with hypoechogenicity; (2) overweight/obese status, hypoechogenicity at USG, and nonsynonymous mutations in TSH-R gene are characterizing features of a large portion of SH children

Ergür et al. [20]^CC^	17 with SH^SG^ 17 without SH^CG^	TSH, TT4, TRH-test, thyroid volume, urinary iodine level, neuropsychological tests, and BMI	7.2 ± 0.89^SG^ 3.6 ± 0.89^CG^	>5.0	1 visit	(1) The SH children obtained significantly lower scores on both the Digit Span subtest of the WISC-R and the Stroop subtests (sensitive to attention); (2) no significant differences found between the SH group and the healthy controls in verbal fluency and encoding tests

Chen et al. [21]^CS^	880 school-aged children (7–18 yrs): 124^SG^ 754^CG^	TSH, FT4, FT3, TPO-Abs, SBP and DBP related to age, gender, height, and 90th percentile (DBP-*Z* and SBP-*Z*)	5.47 ± 1.27^SG^ 2.50 ± 0.84^CG^	>4.2	1 visit	(1) Serum TSH and FT3 (+) correlated with DBP *Z*-score and SBP *Z*-score; (2) DBP-*Z* and SBP-*Z* in subjects with SH were significantly higher than in euthyroid ones; (3) both DBP-*Z* and SBP-*Z* increased linearly in boys with TSH concentrations after adjusting BMI; however a similar linear trend was not observed in girls

Ittermann et al. [22]^CS^	6,435 children aged 3–10 yrs 5,918 adolescents	TSH, FT4, FT3, TPO-Abs, SBP and DBP, and hypertension defined by an increased SBP or DBP using age-, sex-, and height-specific reference values from the KiGGS study	—	—	2 visits	(1) Increased serum TSH levels significantly associated with continuous values of SBP and DBP in children and adolescents; (2) children with increased serum TSH levels had significantly lower pulse pressures and a higher risk of hypertension than euthyroid children

Cerbone et al. [23]^CS,CCL^	49 with SH^SG^ 49 without SH^CG^	TSH, FT3, FT4, TPO-Abs, TG-Abs, SBP, DBP, BMI, waist-to-height ratio, lipid profile, homocysteine, hs-CRP, fibrinogen, adiponectin, and HOMA	6.28 ± 0.2^SG^ 2.75 ± 0.1^CG^	>4.5	38 ± 5	(1) Waist-to-height ratio, atherogenic index, TG to HDL-cholesterol ratio, and homocysteine levels significantly higher and HDL-cholesterol significantly lower in SH subjects compared with controls; (2) glucose, insulin, HOMA index, TG, TC, non-HDL-C, fibrinogen, hs-CRP, and adiponectin concentrations similar in the SH subjects and the controls

Zhang et al. [24]^CC,CS^	24 with SHyperthyroidism 866 euthyroid 29 with SHypothyroidism	TSH, TPO-Abs, thyroid examination BMI, waist circumference, LDL, TG, TC, SBP, DBP, fasting plasma glucose, fasting insulin, and HOMA-IR	<1.01^SHyper^ 1.02–5.72^Euthyroid^ >5.73^SHypo^	>5.73	1 visit	(1) Waist circumference and BMI were significantly greater among adolescents with SHypothyroidism compared with euthyroid subjects; (2) the risk of obesity in the SHypothyroid group was 3.444 times that in the euthyroid group (odds ratio = 3.444, 95% confidence interval (CI): 1.570–7.553); (3) TSH was significantly positively correlated with waist circumference, TC, LDL-C, and TG; (4) TSH level in the metabolic syndrome group was significantly higher than that in nonmetabolic syndrome group

di Mase et al. [25]^CS,CCL^	25 with SH^SG^ 25 without SH^CG^	TSH, FT4, TPO-Abs, TG-Abs, DXA to evaluate lumbar spine BMD, QUS at proximal phalanges of the nondominant hand to assess bone quality, measured as Ad-SoS and BTT	6.39 ± 1.25^SG^ 2.84 ± 0.92^CG^	>4.5	36 ± 3	(1) SH children: normal bone density and structure as assessed by lumbar DXA and phalangeal QUS; (2) both Ad-SoS and BTT normal and comparable to the controls, despite the long-lasting SH

Fallah et al. [26]^CS^	25^SG^ 79^CG^ (MA: 10.46 ± 2.72 yrs)	History, type, and severity of migraine, TSH, and FT4	—	>4.5	1 visit	(1) 24% of 5- to 15-year-old migraineurs had subclinical hypothyroidism; (2) the monthly frequency of headache and the duration of headache were more statistically significant in migraineur children with hypothyroidism

Ekici and Cebeci [27]^CC,CS^	93^CG^ 5^SG^	TSH, FT4, goiter, BMI-SDS, stage of puberty, glucose, TG, HOMA-IR, family history of migraine, and type and severity of migraine	1.96 ± 1.27^CG^ 5.36 ± 1.02^SG^	>5.0	1 visit	(1) None of the investigated parameters (sera FT4, TSH, TC, HDL, LDL, and HOMA-IR) had significant effect on the headache severity score

Cho et al. [28]^R^	254^SG^ 122^CG^	TSH, FT3, FT4, TPO-Abs, TG-Abs, and thyroid USG	—	>5.5	—	(1) No significant difference in the incidence of thyroid disease between children and adolescents with vitiligo and the control group

Onal et al. [29]^CC,CS^	10 newborns out of 98 in vitro babies with SH^SG^, 10 naturally conceived babies^CG^	TRH tests performed in all subjects in^SG,CG^; peak TSH response at the 20th min	0 min of TRH-test: 8.08 ± 1.66^SG^ 7.55 ± 0.80^CG^ 20th min of TRH-test: 55.57 ± 14.31^SG^ 19.37 ± 3.47^CG^	>6.5	1 visit	(1) An exaggerated TSH response to TRH in all of 10 IVF babies but in none of the control babies in study; (2) a significant difference between the two groups with respect to TSH levels at the 20th minute of the TRH stimulation test

Sakka et al. [30]^CC,CS^	106 in vitro children^SG^, 68 randomly selected non-in vitro children^CG^ (aged 4–14 yrs)	TSH, FT3, FT4, TPO-Abs, and TG-Abs	—	>5.0	1 visit	(1) Seven IVF children but none of the controls had persistent elevations of circulating TSH; (2) TSH: significantly higher in the IVF group than in controls; (3) an increased incidence of SH in IVF children compared with controls not attributable to birth weight, gestational age, SGA-AGA status, breastfeeding duration, sex, current age, BMI-SDS, or pubertal status of IVF children or to BMI, thyroiditis, PCOS, GDM, parity, AH, or smoking of their mothers

Kratzsch et al. [31]^R^	1004	TSH, FT3, FT4, T3, T4, t-uptake, TPO-Abs, TG-Abs, WBC, BMI, and gender	—	—	5	(1) BMI-SDS and WBC should also be considered when interpreting TSH and thyroid hormone measurements, whereas gender, TPO-Abs, or TG-Abs play a minor role; (2) BMI-SDS was significantly correlated with T3 and FT3; correlation with FT4 was inversely related; (3) WBC positively correlated with sera TSH, FT4, T3, and T4; (4) FT4 is significantly predicted by age and BMI-SDS and FT3 by and BMI-SDS and WBC; (5) T4 variation depends significantly on age, gender, and WBC while T3 variation was related to age, BMI-SDS, and gender

Ad-SoS: amplitude-dependent speed of sound, BA/CA ratio: bone age/chronological age ratio, BMD: bone mineral density, BTT: bone transmission time, CC: base-control study, CCL: base-control longitudinal study, CG: control group, CRP: C-reactive protein, CS: bross-sectional study, CSL: cross-sectional longitudinal study, DBP: diastolic blood pressure, DBP-*Z*: diastolic blood pressure *Z*-score, DXA: X-ray densitometry, FU: follow-up, HDL: high-density lipoprotein, hs-CRP: high-sensitive C-reactive protein, HOMA-IR: homeostatic model assessment-insulin resistance, IFG-1: insulin-like growth factor 1, IVF: in vitro fertilization, LDL: low-density lipoprotein, MA: mean age, QUS: quantitative ultrasound, R: retrospective, SBP: systolic blood pressure, SBP-*Z*: systolic blood pressure *Z*-score, SG: study group, TC: total cholesterol, TG: triglycerides, TG-Abs: anti-thyroglobulin antibodies, TPO-Abs: anti-thyroid peroxidase antibodies, TRH: Thyrotropin-releasing hormone, UIE: Urinary iodine excretion, and USG: ultrasonography.
